# Advances in nanobody multimerization and multispecificity: from *in vivo* assembly to *in vitro* production

**DOI:** 10.1042/BST20241419

**Published:** 2025-02-07

**Authors:** Mohammed Al-Seragi, Yilun Chen, Franck Duong van Hoa

**Affiliations:** Department of Biochemistry and Molecular Biology, Life Sciences Institute, University of British Columbia, Vancouver, British Columbia, Canada

**Keywords:** affinity, antibody domains, avidity, coiled coils, conjugation, diagnostics, hydrophobic effect, linkers, multimerization, multispecificity, NANOBODIES®, peptidisc, self-assembly, sortase A, therapeutics

## Abstract

NANOBODIES^®^ (Nbs) have emerged as valuable tools across therapeutic, diagnostic, and industrial applications owing to their small size and consequent ability to bind unique epitopes inaccessible to conventional antibodies. While Nbs retrieved from immune libraries normally possess sufficient affinity and specificity for their cognate antigens in the practical use case, their multimerization will often increase functional affinity via avidity effects. Therefore, to rescue binding affinity and broaden targeting specificities, recent efforts have focused on conjugating multiple Nb clones — of identical or unique antigen cognates — together. *In vivo* and *in vitro* approaches, including flexible linkers, antibody domains, self-assembling coiled coils, chemical conjugation, and self-clustering hydrophobic sequences, have been employed to produce multivalent and multispecific Nb constructs. Examples of successful Nb multimerization are diverse, ranging from immunoassaying reagents to virus-neutralizing moieties. This review aims to recapitulate the *in vivo* and *in vitro* modalities to produce multivalent and multispecific Nbs while highlighting the applications, advantages, and drawbacks tied to each method.

## Introduction

Antibodies are Y-shaped molecules produced by B cells synthesized during the onset of an immune response and are pivotal across therapeutic, diagnostic, and industrial regimes [1,2]. At-scale mobilization in these spaces, however, bears room for improvement: antibodies are structurally complex, involving post-translational modifications that demand expensive eukaryotic production systems [3]; their relatively large size (~150 kDa for IgG) may also limit efficient tissue penetration in some clinical settings [4]. Occasionally, monoclonal classical antibodies, such as those derived from hybridomas, or antibody–drug conjugates (ADCs), can induce immunogenic responses, leading to the formation of antidrug antibodies that attenuate therapeutic efficacy [5,6]. To this end, advancements in antibody engineering and development that reconcile these challenges are ongoing.

Over 30 years ago, Hamers-Casterman et al. [7] first discovered and characterized naturally occurring heavy-chain-only antibodies in camel serum [7]. The variable antigen-binding domain (VHH) from these heavy-chain-only antibodies was shown to retain full antigen-binding activity [7], rendering it the smallest naturally occurring antigen-binding fragment identified to date [8]. This standalone structure has since been termed the NANOBODY® (Nb), opening new avenues of research in parallel to the traditional antibody [9]. To their advantage, Nbs are structurally simple, high yielding in *Escherichia coli* expression systems [10,11], thermostable [12,13], and resistant to non-physiological pH levels [14]. Owing to the broadness in the length of their complementary determining region (CDR) relative to human VHHs, Nbs can extend in a ‘finger-like’ fashion to target protein cavities, imparting specificity for unique epitopes and antigen conformers foreclosed to canonical antibodies [15,16]. Remarkable sequence homology between Nbs and human VHHs helps dampen immunogenic responses unwanted in the therapeutic context, with these lower risk profiles having been validated across two Nbs in phase II clinical trials [17]. Given these addresses, researchers have begun adapting Nb-based biologics where antibodies may have been used initially.

While Nbs from immune libraries generally exhibit strong binding affinity, their short serum half-life and specific application in multiplexed assays or low antigen environments may demand enhanced binding and specificity [4]. To rescue their bioanalytical and therapeutic potential, researchers have aimed to multimerize Nbs, thereby leveraging a cumulative affinity effect that improves Nb binding to, and retention with, its cognate antigen via the avidity principle [18–20]. Simultaneously, the conjugation of Nbs with different targeting specificities gives rise to multispecific constructs (hereby called polybodies) with the ability to bind the epitope cognate of every constituent Nb [21]. This approach, crucial for bridging cells, factors, and receptors for combination therapies, enhances target specificity and is gaining significant attention in the development of bispecific antibodies and ADCs [22,23]. Current strategies toward Nb multimerization and multispecificity can be broadly categorized as either *in vivo* or *in vitro* and strategically categorized based on how Nb conjugation takes place. Namely, tandem linking multimerization methods arrange multiple Nb sequences in a linear arrangement [24], multimerization domain methods genetically fuse Nbs to peptides with a natural tendency to self-assembly [25], and chemical methods exploit properties of monomeric Nbs post-translation [26]. Generally speaking, the former two classifications employ *in vivo* production, whereas the latter employs *in vitro* assembly.

To this end, we aim to recapitulate advances in Nb multimerization and multispecificity — both *in vivo* and *in vitro* – while identifying their applications across industrial, therapeutics, and diagnostic sectors.

### Flexible linkers

**Figure 1:**
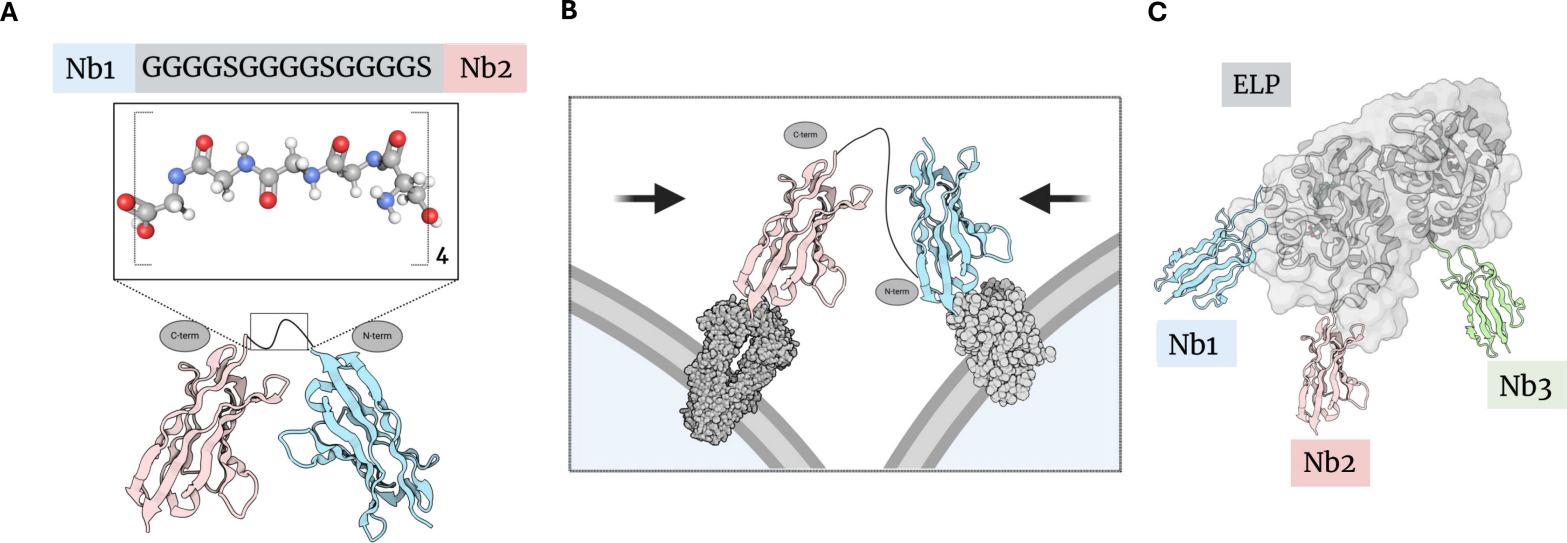
Flexible linkers as a primarily *in vivo* tandem-linking method. **(A)** The (Gly_4_Ser)_3_ linker is amended in sequence between Nb1 and Nb2, affording a bivalent polybody construct. **(B)** The (Gly_4_Ser)_3_ linker formalizes the linkage between two distinct Nb–antigen interactions (bispecific polybody), thereby forming a clamp that proximalizes the two cells along which said antigens occur. **(C)** ELP accommodates three unique Nb structures, though exceptionally by *in vitro* conjugation to afford a trispecific polybody. Nb1, nanobody 1; Nb2, nanobody2.

A ubiquitous *in vivo* approach to procuring polybodies involves the genetic fusion of individual monomeric Nb units in a linear array, resulting in tail-to-head fusion. Formally known as linkers, chains of repeat amino acids are interspaced between two or more Nb sequences. Often employed as a linker is (Gly_4_Ser)_3_, owing to flexibility between fused domains [27] (Figure 1A). Successful implementation thereof has been reported for the synthesis of tetravalent polybodies [28]. Flexible linkers are, however, heterogeneous in form and play a key role in determining the structural dynamics of multimerized constructs [29]. Using (Gly_4_Ser)_3_ as a case study, Li et al. [29] systematically evaluated the structural properties of various linkers used to join Nbs, demonstrating that GlySer linkers exhibit random conformations, whereas linkers rich in Proline residues adopt more rigid and defined structures [29]. Nevertheless, the flexible linker approach can leverage Nb proximity effects. If, for example, two Nbs are fused in tandem to a linker, the corresponding dimerized structure will bind to non-overlapping epitopes, thereby forming a bispecific construct that clamps two antigens together [30] (Figure 1B). In this way, the fusion of two Nb moieties with different targeting specificities enables the proximalization of two different cognate antigens.

In addition to the commonly used (Gly_4_Ser)_3_, the elastin-like protein (ELP) domain has also been employed as a linker for producing multispecific polybodies [31]. Derived from natural elastin, ELP is prized for its flexibility, low immunogenicity, biodegradability, and ability to enhance pharmacokinetics [32]. Schreur et al. [33] coupled ELP – *in vitro* – with three Nbs targeting Rift Valley fever virus (RVFV) [33]. The resulting trispecific polybody demonstrated remarkable virus neutralization efficiency, with an ND_50_ of 0.21 nM (Figure 1C).

It is worth noting that linker-mediated head-to-tail Nb fusion can impose antigen-binding constraints depending on the sequential order of the Nbs. An alternative approach to generating bivalent Nb constructs involves the addition of a C-terminal cysteine residue, or Nb-fusion to cysteine-containing hinge domains, which facilitates covalent dimerization through disulfide bond formation [15,34]. This strategy allows both Nb paratopes to remain free in solution, unlike tail-to-head joining via flexible linkers.

### Antibody domains

**Figure 2:**
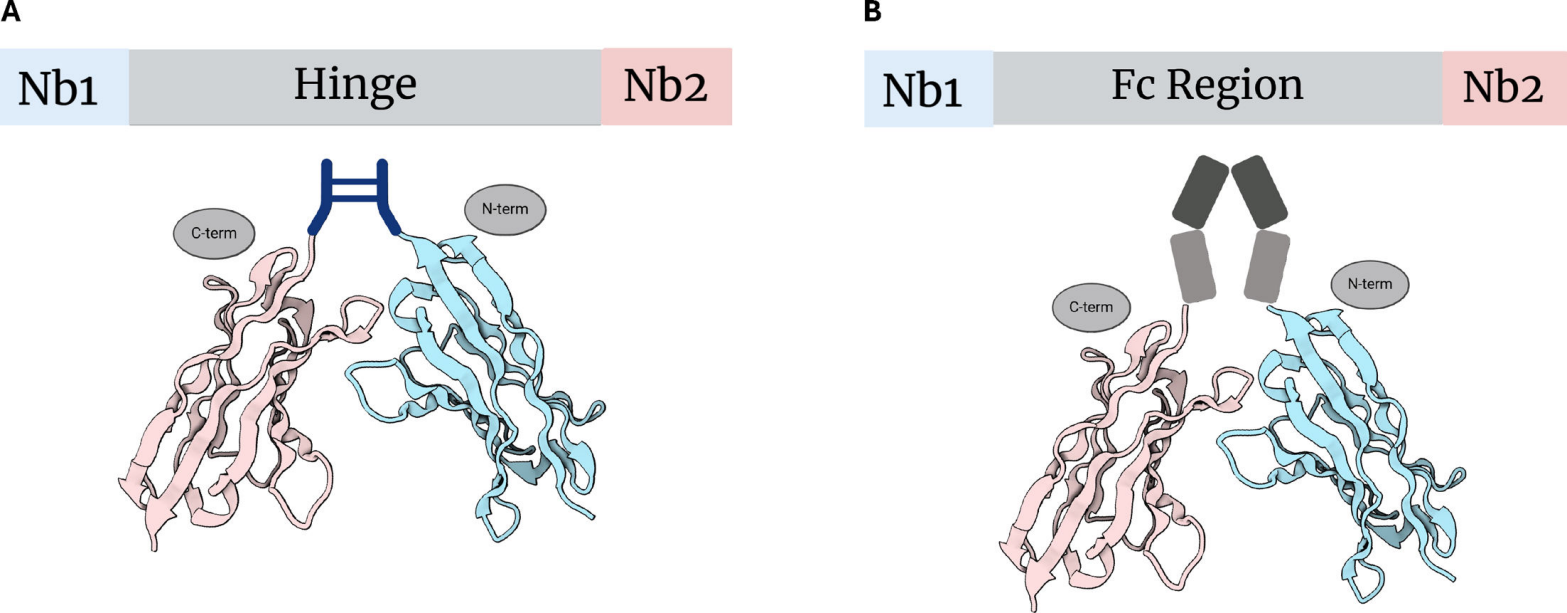
Amending antibody domains as an *in vivo* method. **(A)** The hinge region is amended in sequence between Nb1 and Nb2, affording a bivalent polybody construct. **(B)** The crystallizable Fc region is amended in sequence between Nb1 and Nb2, similarly affording a bivalent polybody construct. Nb1, nanobody 1; Nb2, nanobody 2.

Another common *in vivo* approach towards polybody assembly leverages the functional amendment of domains found in antibodies. One such example involves utilization of the antibody hinge region – specifically the upper hinge of the llama IgG2a – as a linker (Figure 2A). Principally analogous to (Gly_4_Ser)_3_, this upper hinge region has been used to produce bispecific and bivalent polybodies in *E. coli* [34]. Herein, Nbs targeting the hen egg white lysozyme (cAbLys3) and the non-metallo carbapenemase of class A (cAbβLA01) were tethered together via the structural upper hinge of llama IgG2a. To their advantage, this hinge region is protease-resistant in serum and highly flexible. Furthermore, the expression, solubility, and purification of the hinge-tethered polybody were comparable to those of the individual monomeric Nbs [34]. It was observed however that the C-terminal Nb in the dimeric construct exhibited attenuated on-rates, whereas the N-terminal Nb retained its native binding kinetics. In the case of cAbLys3, the C-terminal end of the linker is fused to the N-terminus of the second Nb, resulting in restricted access to the paratope of the second Nb. This restriction likely reduces the k_on_ values of the second Nb, contributing to a 4× greater K_d_ compared with the first Nb. Still, an increase in functional affinity for the bivalent polybody was observed, with an apparent K_d_ falling by a factor of 5 relative to the monomeric cAbLys3 Nb [34].

It is also worth noting that, while Conrath et al. [34] utilized the mouse IgG2a hinge in their study, the more commonly employed hinges are derived from camel or llama IgG2c, or even the human IgA1 hinge. These hinges, which are rich in proline residues, confer greater resistance to protease degradation and adopt a rod-like structure, providing more spatial separation and stability between the two Nbs [35]. For therapeutic applications, the Nb is frequently fused to the hinge and Fc region of human IgG1, employing knob-in-hole technology within the CH3 domain to facilitate the generation of bispecific constructs [36].

Similarly, the antibody Fc region has been used as a framework for genetic fusion of Nb moieties, resulting in tail-to-tail fusion. Cardoso et al. [19] engineered a bivalent anti-influenza virus neuraminidase polybody (N1-VHHm) by fusion of the Nb to the Fc segment of mouse IgG2a, resulting in the N1-VHH-Fc [19] (Figure 2B). Remarkably, the *in vitro* antiviral potency of the N1-VHH-Fc polybodies saw a 30-fold increase relative to their monomeric formats while achieving an IC_50_ in the low nanomolar range. Another use case demonstrated by Gu et al. [37] involved the fusion of two Nbs targeting different domains of the carcinoembryonic antigen-related cell adhesion molecule 5 (CEACAM-5) to a rabbit Fc (rFc) [37]. The resulting bispecific polybodies displayed a roughly one order of magnitude greater affinity for CEACAM-5 compared with the individual Nb fused to rFc, underscoring a significantly enhanced target-binding capacity. Alongside other reports, these experiments have confirmed that multimerization of Nbs heightens functional affinity by introducing avidity [34,38,39].

### Self-assembling coiled coils and peptides

An interesting *in vivo* approach to produce polybodies involves fusion of Nbs to coiled coils with the propensity to self-assemble. Coiled coils are oligomerization motifs commonly found in proteins where 2–7 α-helices spontaneously intertwine by way of Van der Waals interactions, electrostatic attractions, hydrogen bonds, inter-chain disulfide bridges, and hydrophobic effects [40–42]. Of insurgence in this use case is the right-handed coiled coil (RHCC): the C-terminal fragment of the tetrabrachion protein derived from *Staphylothermus marinus* [43]. RHCC folds into a parallel, helical tetramer with four large internal cavities [43] – the lumen of which has been exploited previously to carry anticancer drugs [44]. The coiled-coil domain of cartilage oligomeric matrix glycoprotein (COMPcc) similarly forms five-stranded coiled structures linked together by disulfide bridges with internal hydrophobic pores used for the transport of hydrophobic vitamins [45,46]. Both RHCC and COMPcc share an analogy with the α chain of C4-binding protein (C4bpα) implicated in complement activation, wherein the main human C4bp isoform comprises 7α and 1β secondary structures whose polymerization is driven by the C-terminal region of each constituent chain [47].

**Figure 3:**
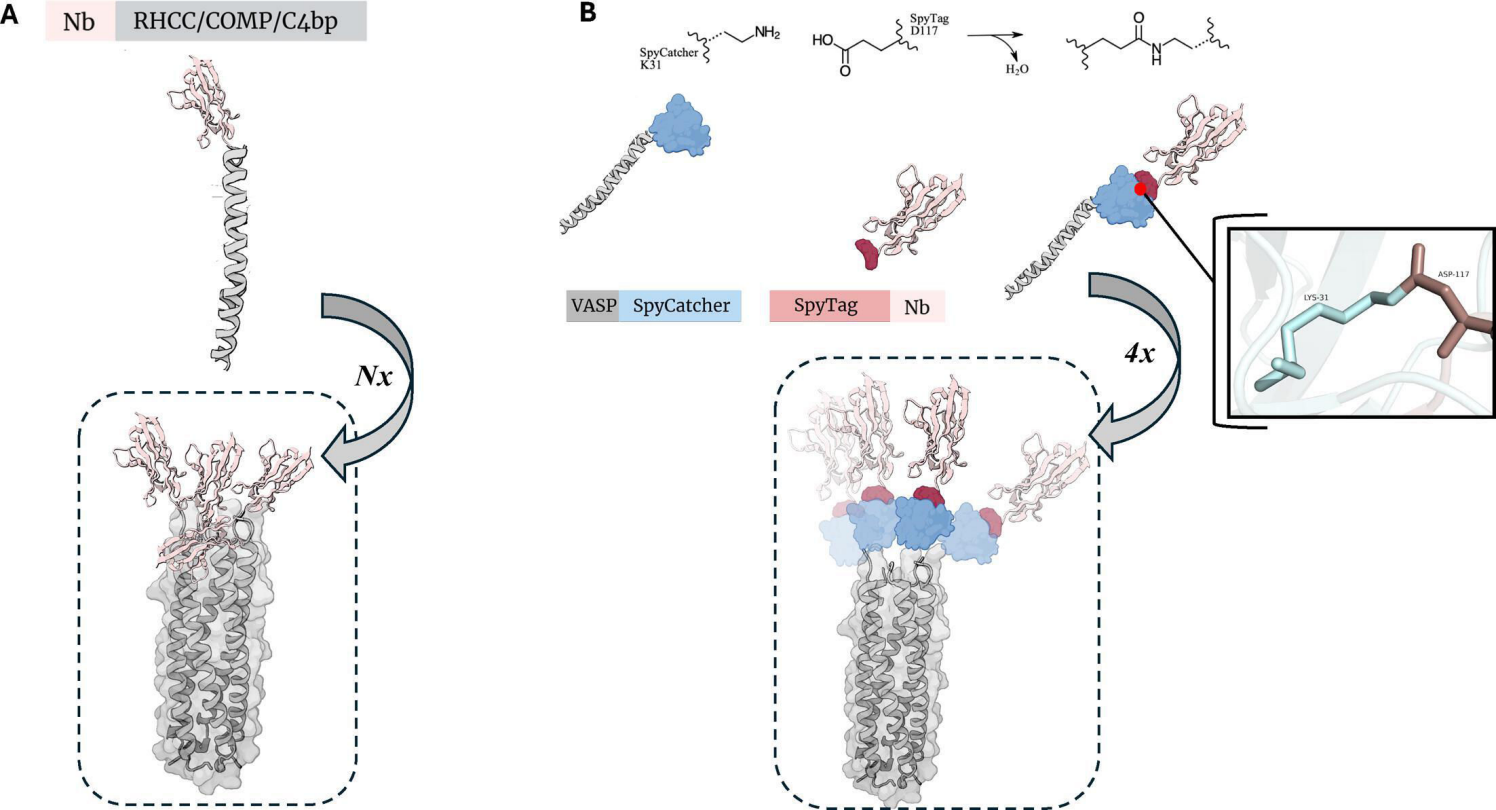
Self-assembling coiled coils as an *in vivo*, multimerization domain method. **(A)** The sequence encoding for a self-assembling coiled coil (e.g., RHCC, COMP, or C4bp) is amended to a sequence encoding for a Nb. The Nb-coiled coil conjugate self assembles with other chimeras in stoichiometry prescribed by the identity of the appended coiled coil, here designated by *N*. **(B)** The SpyTag-SpyCatcher system is employed to conjugate a Nb-to-vasodilator-stimulated phosphoprotein (VASP) conjugate that affords a tetrameric polybody assembly. COMP, cartilage oligomeric matrix glycoprotein; C4bp-binding protein; RHCC, right-handed coiled coil; Nb, nanobody.

In light of this, Wang et al. [25] fused anti-epidermal growth factor receptor (EGFR) Nbs (EG2) to each of these three self-assembling coiled coils − RHCC, COMPcc, and C4bpα – affording tetravalent, pentavalent, and heptavalent polybodies [25] (Figure 3A). They noted that the avidity of the multimeric forms was systematically heightened without perturbing binding specificity and that each polybody construct was able to adapt the correct conformation in the soluble cytoplasmic environment without aggregation [25]. Advantageously, different multivalent states were achieved using different self-assembling peptides, lending some degree of stoichiometric control. Other studies have also reported an enhanced affinity of Nbs following multimerization using these self-associating peptides [48,49].

Exploiting the associative property of coiled coils is not limited to RHCC, COMPcc, and C4bpα, and the assembly of the Nb-coil chimeras need not be constrained to direct genetic fusion of the two elements. Anuar et al. [50] utilized the SpyTag-SpyCatcher system to fuse the pro-apoptotic receptor DR5 Nb (αDR5) to a panel of computationally designed andvasodilator-stimulated phosphoprotein (VASP)/COMPcc/C4bpα-derived coiled coils, affording bivalent to heptavalent DR5 polybodies [50]. Herein, SpyTag was appended to αDR5, while SpyCatcher was attached to the self-assembling coiled coils. When introduced together *in vitro*, the ε nitrogen of the SpyCatcher Lys31 residue spontaneously forms an isopeptide bond with the terminal carboxyl group of the SpyTag Asp117 residue, thus formalizing the Nb-to-coiled coil conjugation [51]. Spontaneous association of parallel, SpyCatcher-conjugated coiled coils together with the introduction of the SpyTag-conjugated Nb drives the assembly of the DR5 polybody (Figure 3B). Of utility in this method is the strong, non-hydrolyzable isopeptide bond and the ability to incubate the phase-two reaction mixture with Spy&Go resin – without isopeptide formation – to recapture unconjugated αDR5-SpyTag, such that only the αDR5-coiled coil oligomers are recovered. Unfortunately, attempts at multimerizing anti-DR5 Nbs to this point had resulted in strictly linear arrangements that did not mimic the clustering of endogenous peptide ligands to the DR5 receptor [52–54], emphasizing that conformation and three-dimensional shape are just as important a consideration as valency and Nb copy number in the procurement of biologically functional polybody constructs.

Besides coiled coils, other self-associating peptides have also been employed for Nb multimerization. Diestch et al. [55] discovered that a 31-amino acid peptide derived from the p53 tetramerization domain, with two mutations (E343K/E346K, referred to as E3), could be fused to the N-terminal of an anti-GFP Nb, promoting stable dimer formation with enhanced binding properties [55]. A notable advantage of using the E3 peptide-modulated Nb dimer is its specificity and improved signal-to-noise ratio *in cellulo*, compared with the tandem-linked dimer [55].

### Self-assembling subunits

**Figure 4:**
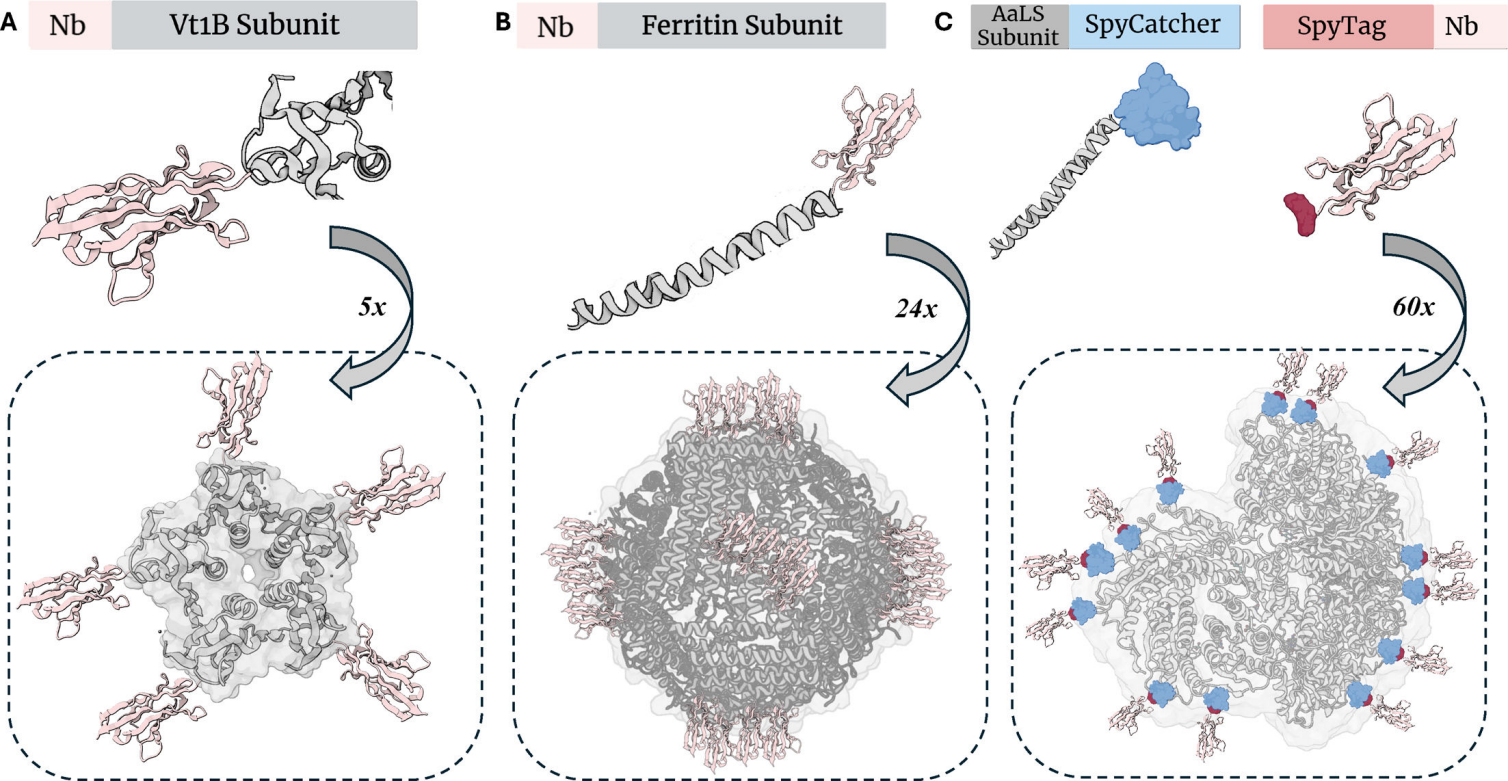
Self-assembling subunits as an *in-vivo*, multimerization domain method. **(A)** The sequence encoding for a VT1B subunit is amended to a sequence encoding for an Nb, lending to spontaneous pentavalent assembly. **(B)** The sequence encoding for a ferritin subunit is amended to a sequence encoding for an Nb, lending to spontaneous 24-mer assembly. **(C)** A SpyCatcher-AaLS chimera is introduced to a SpyTag-Nb chimera, lending to a 60-mer assembly. Nb, nanobody; VT1B, verotoxin 1B subunit.

Like approaches exploiting self-assembling secondary structures – as is the case with coiled coils – entire protein subunits with similar properties have been adapted for the development of novel polybody constructs [37,56]. One such example involves the *E. coli* O157:H7 verotoxin 1B subunit (VT1B): a pentameric, toroidal structure with peripheral exposure of its N and C termini [57–59]. Owing to its ability to spontaneously assemble into a pentamer, Zhang et al. [56] fused the VT1B with parathyroid hormone (PTH)-targeting Nb to afford a pentavalent polybody [56] (Figure 4A). While pentamerization of antibodies has also been explored using the homopentameric cartilage oligomeric matrix protein (COMP), the 20 Å-diameter assembly was thought to yield a particle with ineffective geometry [60]. This, however, was not a concern with the pentavalent anti-PTH polybody, wherein the peripheral position of the N and C termini enabled optimal presentation of the five VT1B-proximalized Nb monomers [56]. Accordingly, antigen-binding profiles revealed that the pentameric VT1B-Nb chimera bound immobilized the PTH peptide more effectively than standalone Nbs, confirming that VT1B fusion to monomeric Nbs does not significantly alter bind site accessibility [56]. Advantageously, the use of the VT1B granted facile expression of the VT1B-Nb chimera without aggregation while also allowing the pentameric polybody to confer excellent thermostability and protease resistance [56]. Since its establishment in 2004, the VT1B fusion strategy has been applied in diagnostics, including, for example, the FITC-labeled, pentavalent CEACAM-5 polybody in immunoassays [37].

Ferritin – a spherical, iron-storing protein composed of a self-assembled 24-subunit protein cage [61] – has also been employed as a structural framework for polybody development [62]. Of the octahedral symmetry, ferritin is made from 24 subunits each comprising four long helices (α−δ), a fifth short helix (ε), and a long extended loop between the β and the γ helices [63]. To its advantage, ferritin is conducive to high yield in *E. coli* expression systems [64], is thermostable owing to its many salt bridges and hydrogen bonds, and demonstrates remarkable *in vivo* biocompatibility [65,66]. Using ferritin from the hyperthermophilic arachaea *Pyrococcus furiosus,* Fan et al. [62] produced a 24-mer from monomeric anti-H5N1 virus Nb [62], affording a structure termed the fenobody. Transmission electron microscopy revealed that the fenobody, much like that of the native ferritin protein, preserved a cage-like assembly [62] (Figure 4B). Due to an increase in size, the fenobody also gained a 10× extension in serum half-life and a 360× increase in apparent affinity for the H5N1 virus using a murine model. Recently, the fenobody has been utilized as the capture antibody in sandwich ELISA assays and when combined with RANbody (an Nb-fused reporter) demonstrated heightened sensitivity and specificity compared with commercially available assays for detecting Newcastle disease virus (NDV) [67].

Like ferritin, lumazine synthase (LS) has been used as a structural framework for Nb display and, consequently, polybody assembly [68]. LS is uniform in size, structurally symmetric, and thermally stable with a melting point of 120°C [69]. Unlike ferritin, however, LS is a hallowed dodecahdedron of 60 identical subunits with the potential to thus accommodate 60 Nb copies. Exploiting this property, Lu et al. [68] used the SpyTag-SpyCatcher system to fuse *Aquifex aeolicus* LS (AaLS) protein with anti-omicron Nbs, thereby affording a corresponding anti-omicron 60-mer polybody [68] (Figure 4C). Whereas the monomeric B-B2 Nb demonstrated an IC_50_ of 1.658 μg/mL against the B.1.1.529 omicron pseudovirus, the AaLS 60-mer chimera achieved an IC_50_ of 0.653 μg/mL, maintaining that LS-induced multimerization is a viable platform for ameliorating Nb neutralization potency [68].

### Enzymatic and chemical mediation

**Figure 5:**
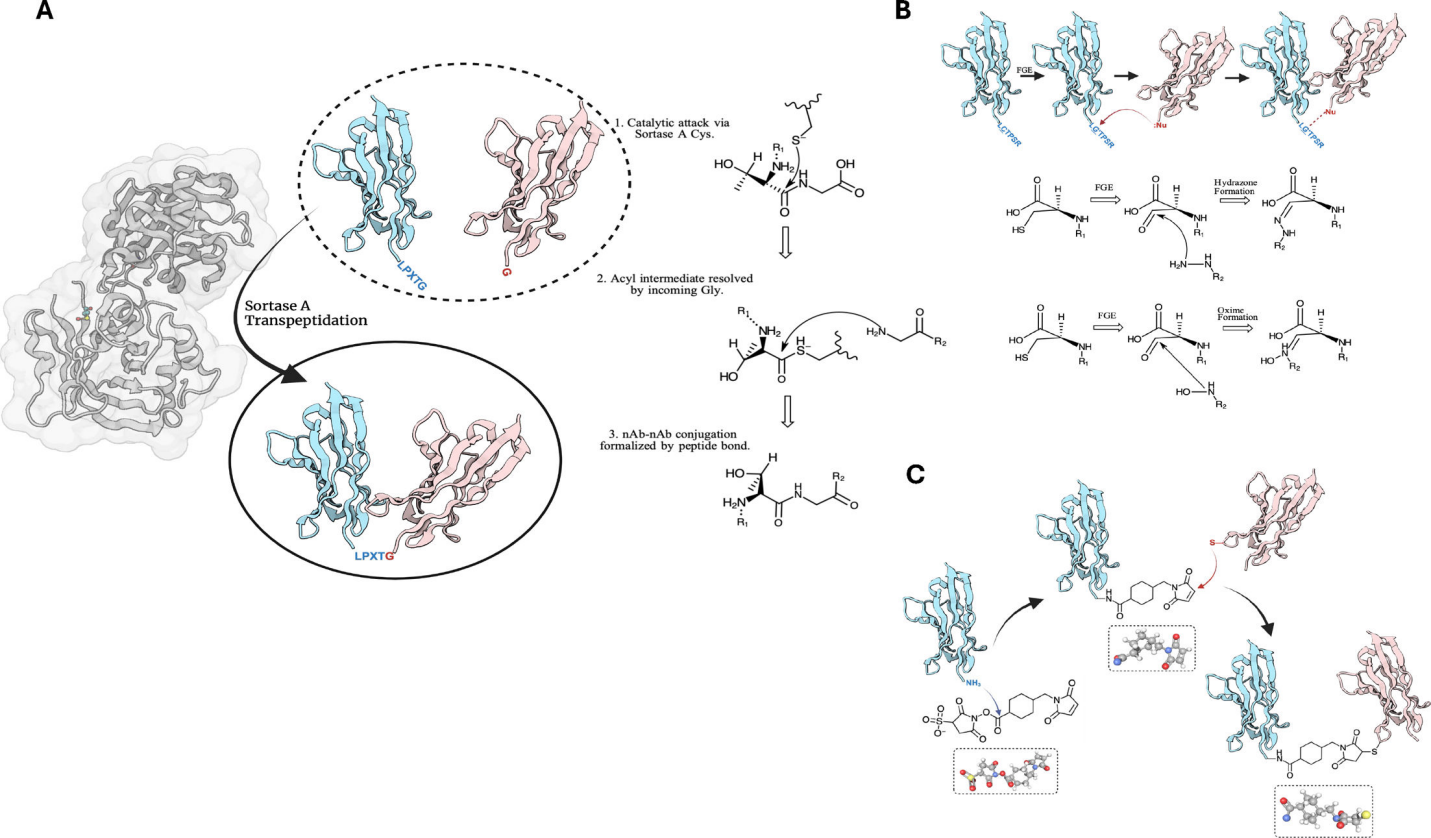
Chemical and enzymatic mediation as an *in vitro* method. **(A)** Sortase A transpeptidation in theory can formalize Nb–Nb conjugation in the presence of an LPXTG tag and an attacking glycine residue through an acyl intermediate. **(B)** FGE in theory can catalyze Nb–Nb conjugation in the presence of an LCTPSR tag and a biorthogonal nucleophilic carrier. **(C)** Sulfo-SMCC in theory can cross-link a primary amine-carrying Nb and sulfhydryl-carrying Nb via thioether and amide bonds. FGE, formyl-glycine generating enzyme; Nb, nanobody.

The pivot toward *in vitro* polybody assembly is paramount for at-scale, industrial production and has been one grounded in high throughput enzymatic and chemical methodologies. Enzyme-mediated ligation of Nbs has been approached in a variety of ways, but of prevalence are those employing a form of transpeptidase activity. Sortase A, for example, is a transpeptidase derived from *Staphylococcus* that endogenously catalyzes protein tethering to the peptidoglycan coat of the bacterial cell wall [70,71]. Obeng et al. [20] used Sortase A transpeptidation to produce anti-mCherry Nb-biotin and anti-SARS-CoV-2-S Nb-biotin conjugates – seven or more of which were docked to a single streptavidin-coated quantum dot to afford at least a heptavalent polybody with highly uniform assembly [20]. To drive Sortase-A-mediated conjugation, one protein must bear a C-terminal LPXTG tag and another must bear a primary amine-containing moiety (commonly Gly) [72]. Mechanistically, the catalytic cysteine residue of Sortase A attacks the peptide bond between the threonine and the glycine tag residues on the first protein to afford a thioacyl intermediate; the N-terminal glycine of the second protein resolves this tetrahedral intermediate and formalizes protein–protein conjugation, concomitant with the release of the bispecific product from the Sortase A active site [72] (Figure 5A).

Another similar-spirited enzymatic approach involves the formyl-glycine generating enzyme (FGE). FGE works by catalyzing the oxidation of a cysteine residue in the LCTPSR sequence motif (or more broadly, CXPXR) [73], affording formylglycine from the sulfhydryl group [74]. This aldehyde moiety is available for site-specific reactions with various orthogonal nucleophiles, including hydrazide or aminooxy-functionalized molecules via chemoselective ligation [75,76], rendering it of utility for conjugation with fluorescent dyes, biotin, or chromatography resins [77] (Figure 5B). Although this is not an example of bispecificity in the traditional sense where one Nb is fused to another, this methodology has been applied previously in protein–protein conjugation and, thus, bears potential for the procurement of polybody constructs [73,78,79].

Even more detached from *in vivo* methodologies and biological systems at large are those that employ cross-linking reagents. The maleimide-based sulfo-SMCC (sulfosuccinimidyl-4-(N-maleimidomethyl)cyclohexane-1-carboxylate) linker has been used to fuse anti-endothelial growth factor receptors 2 (VEGFR-2) Nbs to truncated diphtheria toxin for targeted cancer cell apoptosis [80]. Similarly, the sulfo-SMCC reagent has been used to cross-link four copies of anti-carcinoembryonic antigen (CEA) Nbs to an amino-modified CdSe–ZnS quantum dot, affording up to a tetravalent polybody assembly [81]. Here, sulfo-SMCC reacts with a primary amine on one protein and a cysteine sulfhydryl group on another, linking both proteins together via thioether and amide bonds [26] (Figure 5C). This technique takes advantage of an unpaired cysteine residue engineered in the VHH, thereby allowing for site-specific conjugation at either the N or C terminus.

The principle of using a medium, such as the CdSe–ZnS quantum dot, to tetramerize Nbs draws analogy with other robust multimerization techniques. For example, the introduction of a biotinylation tag, such as the Avi-tag, at the C-terminus of the Nb offers a straightforward method for site-specific biotinylation using the BirA enzyme [82]. This approach facilitates the immobilization of Nbs onto streptavidin-coated surfaces, enabling the generation of tetravalent Nb constructs with all paratopes oriented toward the solution, thereby maximizing their antigen-binding potential.

### Transmembrane segment clustering with membrane mimetics

**Figure 6:**
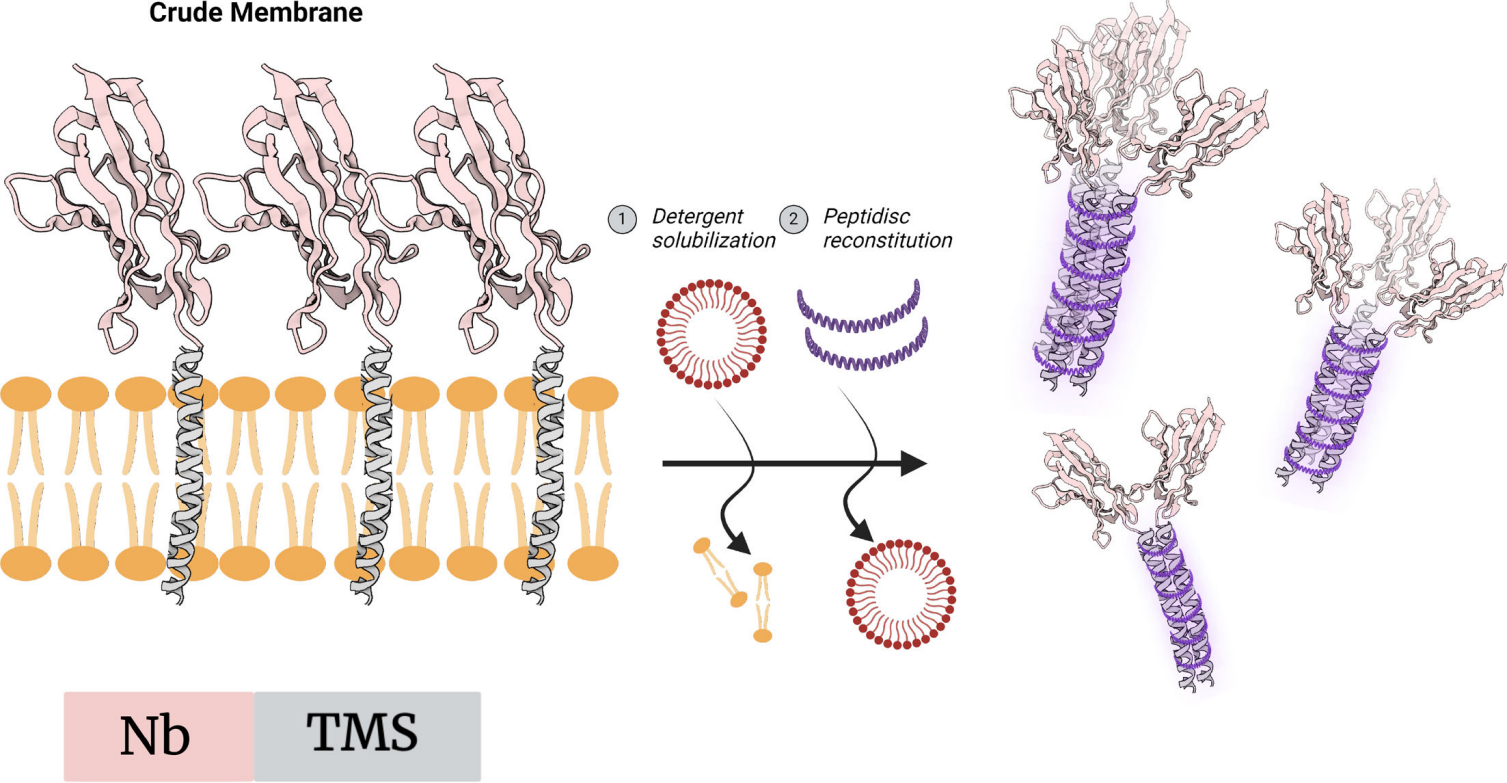
Transmembrane segment appendage with membrane mimetics as an *in vitro* method. TMS–Nb chimeras are expressed in the cell membrane and can be liberated using mild detergent. The amphipathic peptidisc mimetic stabilizes hydrophobic interactions at TMS junctions. Nb, nanobody; TMS, transmembrane segment.

An almost insurmountable challenge in the *in vitro* procurement of protein–protein conjugates is one grounded in kinetics: the hope is that proteins couple together at industrially relevant rates even at lower applied concentrations [83]. In search of a more rapid driving force, Chen et al. [84] appended a single C-terminal transmembrane segment (TMS) to anti-GFP and anti-HSA Nbs, allowing for their expression in the membrane bilayer [84]. Owing to the hydrophobicity of the TMS, these artificial single-pass membrane proteins tend to quickly associate together at common loci where the TMS regions are sequestered from the aqueous environment. Exploiting this hydrophobic property, Chen et al. [84] employed the amphipathic membrane mimetic peptidisc to stabilize chimeric anti-GFP and anti-HSA polybody constructs (Figure 6). Herein, the internal hydrophobic face of the peptidisc mimetic joins Nbs at their TMS–TMS junctions, while the external hydrophilic face maintains water solubility. The polyhistidine tag on the TMS fusion protein enables immobilization and purification while also providing spatial proximity for protein–protein interaction. With further functionalization, the transient hydrophobic interaction holding the polybody assembly together could then be fixed using a glutaraldehyde cross-linking step that takes advantage of the available lysine residues in the peptidisc primary sequence. Employing this methodology, Chen et al. [84] were able to assemble a multivalent anti-GFP polybody with observed avidity effects upon oligomerization. Moreover, the use of the multivalent anti-HSA polybody enhanced ELISA detection sensitivity by 32-fold. To our knowledge, this method is the first of its kind to generate multivalent and multispecific Nbs by leveraging properties derived from membrane proteins. As it stands currently, stoichiometry using this methodology cannot be controlled, with Nb ratio and polybody size remaining heterogeneous.

## Methods summary

Modalities toward polybody assembly are diverse. The expansive toolbox for procurement of multivalent and/or multispecific polybody constructs allows researchers to opt for the methodology that best suits their experimental needs. Recapitulated in Table 1 are the polybody platforms outlined in this review paper, their categorizations, with advantages and disadvantages inherent to each method.

**Table 1:** Summary of modalities toward Nb multimerization and multispecificity, including classifications, examples, advantages, and limitations inherent to each method.

Nature	Class	Technique	Feature	Example	Advantages	Limitations
*In vivo*	Tandem linking	Linker	Gly_4_Ser_3_	Nb7-14; Nb28_3_; Nb28_4_ [28,85]	Highly flexible	Expression yield decreases as the copy number increases [28,29]; may affect structural integrity and binding affinity of Nbs if many linkers are applied in tandem [86]
			ELP	Rv104-elp-rv107-elpabd-rv150 [33]	Flexible, low immunogenicity, biodegradable, and can enhance pharmacokinetics [32]	Insoluble expression of protein requires 6 M guanidine hydrochloride as a denaturant followed by refolding
		Antibody domain	IgG hinge	cAbβLA01-cAblys3 (bispecific) cAblys3-cAblys3 (bivalent) [34]	Flexible and protease degradation resistance	Susceptible to proteolytic cleavage [87]
			IgG Fc	N1-VHH-Fc (bivalent) [19] Nb1–Nb2-rFc (bispecific) [37]	Fc region retained for potential Fc receptor interaction	Requires eukaryotic expression system; may trigger unintended antibody-dependent cellular cytotoxicity [88].
	Multimerization domain	Self-Assembling coiled coils	RHCC	EG2-RHCC [25]	Has potential to be used as a drug carrier owing to an internal storage cavity	Lack of the interchain disulfide bond instability
			COMPcc	EG2-COMPcc, oPent-αDR5, MT1COMP (COMBODY) [25,50]	Capable of storage and delivery of vitamin D and other hydrophobic compounds	Sequence-dependent production yield [48]
			C4bpα	EG2-C4bpα, oHept-αDR5, Nb28-C4bpα [25,50]	Maximum number of Nb copies among coiled-coil approaches	The protein may be insoluble and requires 8 M urea as a denaturant followed by refolding
			VASP	oTet-DR5 [50]	Efficient and modular	Risk of unwanted immunogenicity
			Computationally designed coiled coil	oDi-αDR5, oTri-αDR5, oHex-αDR5 [50]	Efficient and modular.	The protein maybe insoluble and requires 8 M urea as a denaturant followed by refolding (oHex)
			P53 tetramerization domain	nano-eGFP-E3 [55]	Allow the assembly for homodimer or heterodimer, thermostable.	Risk of unwanted immunogenicity
		Self-Assembling subunits	VT1B	1V5 (pentabody), Nb3-VT1B [57,37]	Nbs can be fused to both termini of VT1B, making it possible to produce monospecific and bispecific polybodies.	Concern on potential toxicity for in vivo applications.
			Ferritin	H5N1–fenobody [64]	Has an internal storage cavity for small molecules/drugs.	Fenobody may be insoluble [67].
			Lumazine synthase	LS-B1-4 [68]	Thermostable.	Large assembly may impede pharmacokinetics and tissue penetration.
*In vitro*	Chemical and enzymatic mediation		Sortase A	mCherry QNC, SARS-CoV-2-S–avidin NAC [20]	Site-specific biotin conjugation	Poor catalytic efficiency; reversible reaction; requires large excess of enzyme and nucleophile-bearing protein [89]
			FGE	Nb-dye/biotin/resin [75]	Highly site-specific	Yield heterogenous conjugates; portions of the tag may attenuate conjugate stability [90]
			Sulfo-SMCC	Anti-VEGFR2–diptheria toxin [80]	Stable covalent linkage	Yield heterogenous conjugates; bias for larger conjugates [91]; irreversible unfolding if buried scaffold cysteines are conjugated [92]
			TMS	GFP polybody, HSA polybody, GFP/HSA bispecific polybody, fluorescent HSA polybody [84]	Peptidisc can be functionalized	Yield heterogenous conjugates; stoichiometry is not controlled

## Conclusion

Advancements in Nb multimerization and multispecificity offer promising solutions to overcome the inherent limitations of monomeric Nbs while providing new avenues of application foreclosed to the canonical antibody. By employing a diverse set of *in vivo* and *in vitro* methodologies – including flexible linkers, antibody hinge domains, self-assembling coiled coils, and chemical conjugation techniques – researchers have engineered constructs with improved functional affinity, specificity, and stability. These efforts have inspired the creation of multivalent and multispecific constructs capable of tackling increasingly sophisticated tasks in diagnostics, therapeutics, and industrial landscapes. By leveraging principles such as avidity, modularity, and precise molecular assembly, the field continues to push the boundaries of what can be achieved with these unique biomolecules.

Still, challenges such as production yield optimization and maintenance of structural integrity remain across these multimerization platforms, urging that exploration and refinement in this field are ongoing. Future research must prioritize universal and scalable approaches for Nb engineering that balance modularity, stability, and ease of production. Equally important is the high-resolution structural characterization of multivalent and multispecific Nbs, which will provide critical insights into their binding mechanisms and facilitate rational design routes. With their unique properties and expanding capabilities, multivalent and multispecific Nbs are poised to profoundly redefine biotherapeutic and diagnostic endeavors.

**Perspectives**

1. Nb engineering is a rapidly evolving field of research focused on ameliorating therapeutic, diagnostic, and industrial applications by offering alternatives to conventional antibodies with respect to size, stability, and production facility.
2. Current strategies aim to circumvent the weaker affinity of Nbs by leveraging the avidity effect, whereby identical or unique Nbs are conjugated together. Modalities toward Nb multimerization and multispecificity may be *in vivo* or *in vitro* by nature and collectively work to improve affinity, half-life, and functional versatility.
3. While functional assays have been performed on multimerized Nbs, achieving high-resolution structures thereof is crucial for understanding binding mechanisms and optimizing performance. Universal approaches toward constructing multimeric and multispecific Nbs with modularity and compositional control are highly limited, warranting comprehensive evaluations not restricted to simple one-to-one method comparison.

## Abbreviations

ADCs: antibody–drug conjugates
CDR: complementary determining region
CEA: carcinoembryonic antigen
CEACAM-5: carcinoembryonic antigen-related cell adhesion molecule 5
COMP: cartilage oligomeric matrix protein
COMPcc: cartilage oligomeric matrix glycoprotein
EGFR: anti-epidermal growth factor receptor
ELP: elastin-like protein
FGE: formyl-glycine generating enzyme
LS: lumazine synthase
NDV: Newcastle disease virus
Nbs: NANOBODIES®
PTH: parathyroid hormone
RHCC: right-handed coiled coil
RVFV: Rift Valley fever virus
TMS: transmembrane segment
VASP: vasodilator-stimulated phosphoprotein
VHH: variable antigen-binding domain
